# Salt Stress Represses Soybean Seed Germination by Negatively Regulating GA Biosynthesis While Positively Mediating ABA Biosynthesis

**DOI:** 10.3389/fpls.2017.01372

**Published:** 2017-08-10

**Authors:** Kai Shu, Ying Qi, Feng Chen, Yongjie Meng, Xiaofeng Luo, Haiwei Shuai, Wenguan Zhou, Jun Ding, Junbo Du, Jiang Liu, Feng Yang, Qiang Wang, Weiguo Liu, Taiwen Yong, Xiaochun Wang, Yuqi Feng, Wenyu Yang

**Affiliations:** ^1^Key Laboratory of Crop Ecophysiology and Farming System in Southwest China, Sichuan Engineering Research Center for Crop Strip Intercropping System, Institute of Ecological Agriculture, Sichuan Agricultural University Chengdu, China; ^2^Key Laboratory of Analytical Chemistry for Biology and Medicine (Ministry of Education), Department of Chemistry, Wuhan University Wuhan, China

**Keywords:** salinity, seed germination, abscisic acid, gibberellin, soybean

## Abstract

Soybean is an important and staple oilseed crop worldwide. Salinity stress has adverse effects on soybean development periods, especially on seed germination and post-germinative growth. Improving seed germination and emergence will have positive effects under salt stress conditions on agricultural production. Here we report that NaCl delays soybean seed germination by negatively regulating gibberellin (GA) while positively mediating abscisic acid (ABA) biogenesis, which leads to a decrease in the GA/ABA ratio. This study suggests that fluridone (FLUN), an ABA biogenesis inhibitor, might be a potential plant growth regulator that can promote soybean seed germination under saline stress. Different soybean cultivars, which possessed distinct genetic backgrounds, showed a similar repressed phenotype during seed germination under exogenous NaCl application. Biochemical analysis revealed that NaCl treatment led to high MDA (malondialdehyde) level during germination and the post-germinative growth stages. Furthermore, catalase, superoxide dismutase, and peroxidase activities also changed after NaCl treatment. Subsequent quantitative Real-Time Polymerase Chain Reaction analysis showed that the transcription levels of ABA and GA biogenesis and signaling genes were altered after NaCl treatment. In line with this, phytohormone measurement also revealed that NaCl considerably down-regulated active GA_1_, GA_3_, and GA_4_ levels, whereas the ABA content was up-regulated; and therefore ratios, such as GA_1_/ABA, GA_3_/ABA, and GA_4_/ABA, are decreased. Consistent with the hormonal quantification, FLUN partially rescued the delayed-germination phenotype caused by NaCl-treatment. Altogether, these results demonstrate that NaCl stress inhibits soybean seed germination by decreasing the GA/ABA ratio, and that FLUN might be a potential plant growth regulator that could promote soybean seed germination under salinity stress.

## Introduction

Salinity is one of the most important abiotic stresses in the world. It has adverse effects on almost all development stages during the plant life-cycle, including seed germination, seedling establishment and development, vegetative and reproductive growth, and crop survival and yield (Zhu, 2016). More than 45 million hectares of agricultural land have been damaged by salt worldwide, and NaCl stress is an important salinity stress (Munns and Tester, 2008). Over the past few decades, numerous studies have demonstrated that high soil salinity content significantly affects crop growth and development through many diverse pathways, including water stress, nutritional disorders, ion toxicity, oxidative stress, alterations to metabolic processes, cell membrane disorganization, and reduced cell expansion and division (Hasegawa et al., 2000; Munns, 2002; Zhu, 2002, 2016; Teige et al., 2004; Hanin et al., 2016). Therefore, the physiological and molecular mechanisms through which plants adapt to salt stress need further investigating and the results could lead to improvements in agricultural production worldwide, especially on saline land.

Soybean (*Glycine max* (L.) Merrill) is an important and staple oil crop worldwide, and is used as a plant oil and protein resource by humans. However, salinity stress has significantly decreased soybean yield by inhibiting seed germination and post-germinative growth. Several key regulators, including *GmSALT3, GmMYB76, GmMYB92, GmMYB177, GmbZIP132*, and *MiR172C*, have been documented, and their related-mechanisms were investigated (Liao et al., 2008a,b; Guan et al., 2014; Wang et al., 2014; Kan et al., 2016; Li et al., 2016; Liu et al., 2016). High and uniform germination and emergence in the field are the key determinants of soybean yield, especially under salt stress conditions. Salinity represses soybean seed germination (Zhang et al., 2014; Kan et al., 2016), but the precise mechanisms underlying the inhibition effect of salt on soybean seed germination are only partially understood. Further, potential plant growth regulators need to be developed to promote soybean seed germination and emergence under salinity stress conditions.

Seed germination and post-germinative growth are critical stages during the plant life-cycle. The phytohormone abscisic acid (ABA) delays seed germination whereas gibberellin (GA) promotes this process, and both hormones are key regulators involved in seed germination processes (Finkelstein et al., 2008; Graeber et al., 2012; Nee et al., 2016; Shu et al., 2016b). Consistently, mutants with dysfunctional ABA and GA biosynthesis, and catabolic or signaling transduction pathways always show an altered germination phenotype. In *Arabidopsis*, the ABA biosynthesis mutants *nced6, nced5, nced3*, and *aba2* (Seo et al., 2006; Frey et al., 2012), and ABA signaling mutants *abi3* (Koornneef et al., 1989), *abi4* (Shu et al., 2013), and *abi5* (Piskurewicz et al., 2008) had a faster germination phenotype compared to wild type, whereas overexpression of *Nicotiana ABA2* or *Arabidopsis ABI4* resulted in a delayed-germination phenotype (Frey et al., 1999; Shu et al., 2013). However, the seeds of GA-deficiency mutants, *ga1* and *ga2*, failed to germinate unless there was an exogenous supply of GA (Lee et al., 2002; Shu et al., 2013), while the mutants that are defective in GA2-oxidases (GA2ox), which deactivate bioactive GA, have a faster germination phenotype (Yamauchi et al., 2007). Further, the ratio between the levels of active GA/ABA is also a key determinant of seed germination (Shu et al., 2015, 2016b; Meng et al., 2016). Taken together, both ABA and GA levels, along with signaling play important roles in the regulation of seed germination.

Several abiotic stress conditions, including drought and salinity, lead to the up-regulation of ABA, which in turn elicits diverse adaptive responses in plants (Zhu, 2002, 2016). Osmotic stress–induced ABA accumulation is largely dependent on the activation of biosynthesis and the inhibition of degradation pathways (Zhu, 2002). Previous studies revealed that applying a salt treatment up-regulated the transcription levels of *ZEP* and *NCED* genes, which encode the key enzymes involved in the ABA biosynthesis pathway (Xiong et al., 2002; Galle et al., 2013). Furthermore, some ABA biosynthetic mutants such as *aba2* seeds germinate earlier than the wild type under salt stress conditions (Gonzalez-Guzman et al., 2002), and *ABA2* transcript can also be upregulated by prolonged salt treatments (Lin et al., 2007). On the other hand, salinity negatively regulates GA biosynthesis during the seed germination and seedling development stages (Shakirova et al., 2003; Horvath et al., 2015; Zhang et al., 2016). A recent study showed that ABA and GA biosynthesis were also involved in seed heteromorphism in *Suaeda salsa* under salt stress conditions (Li et al., 2015). However, the relationship between salt stress and GA still needs further investigation, especially during the soybean seed germination processes.

This study shows that exogenous NaCl treatment inhibits soybean seed germination by mediating the ABA and GA biogenesis pathways, and importantly, our evidences demonstrate that an ABA biogenesis inhibitor, fluridone (FLUN), might be a potential plant growth regulator that could be used to enhance soybean seed germination under salinity stress conditions. The quantitative Real-Time Polymerase Chain Reaction (qRT-PCR) analysis revealed that the transcription levels of ABA and GA biogenesis genes were altered by NaCl application. Consistently, further phytohormone quantification revealed that NaCl remarkably down-regulated active GA_1_, GA_3_, and GA_4_ levels, whereas ABA content was up-regulated; and consequently the ratios for GA/ABA (including GA_1_/ABA, GA_3_/ABA, and GA_4_/ABA) were significantly decreased. Taken together, it can be concluded that salt stress delays soybean seed germination by negatively regulating GA while positively mediating ABA biogenesis and that this consequently leads to a reduction in the GA/ABA ratios. Last but not least, FLUN can be used as a potential chemical to promote soybean seed germination and post-germinative growth under salinity stress conditions.

## Results

### Exogenous NaCl Application Delays Soybean Seed Germination

**FIGURE 1:**
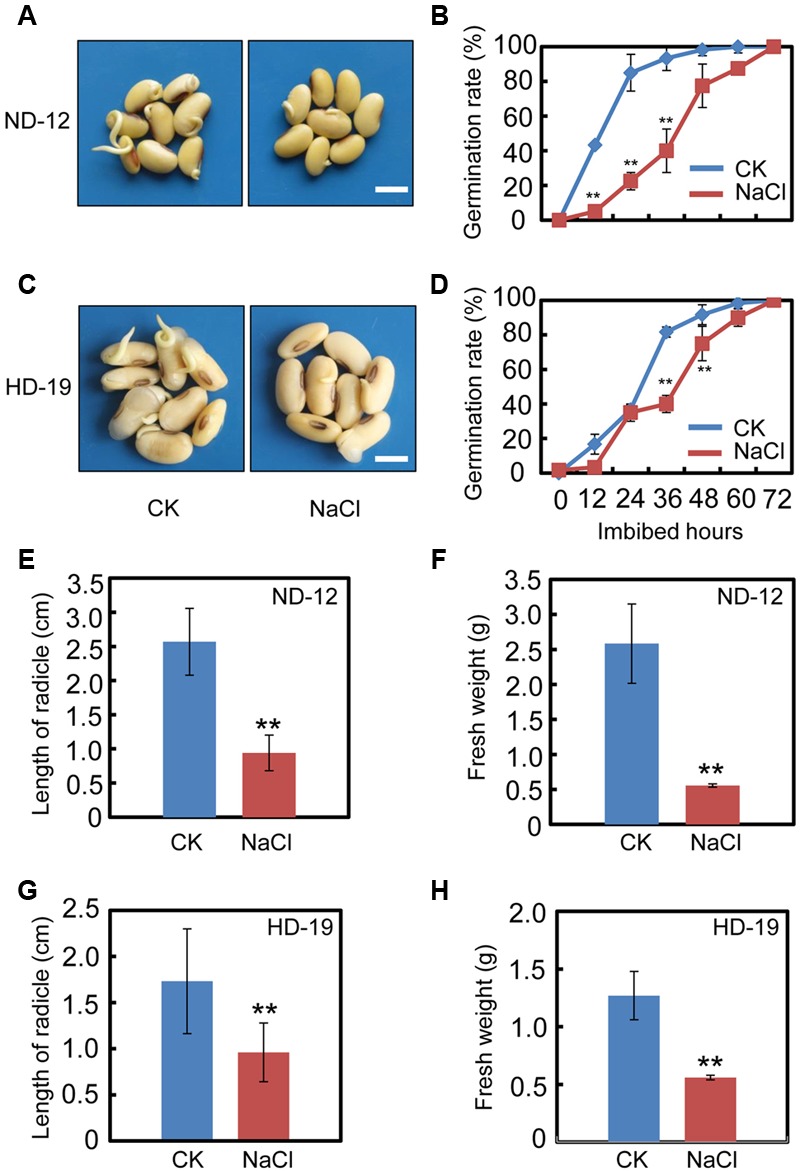
NaCl inhibits soybean seed germination. Healthy soybean seeds (cultivars ND12 and HD19) were incubated on two layers of filter paper in Petri dishes. The concentration of NaCl used was 150 mM, and the equivalent ultrapure water was employed as control (CK). **(A)** The representative images (36 h after sowing) are shown for cultivar ND12. **(B)** Quantitative analysis of germination rates is shown for ND12. **(C)** The representative images (36 h after sowing) are shown for cultivar HD19. **(D)** Quantitative analysis of germination rates is shown for HD19. **(E,F)** Radicle length and fresh weight of germinated soybean seeds were measured for cultivar ND12. **(G,H)** Radicle length and fresh weight of germinated soybean seeds were measured for cultivar HD19. Bar = 10 mm. The average percentages of four repeats ± standard error were shown. Student’s *t*-test assay was employed for statistical analysis, and ^∗∗^ difference is significant at the 0.01 level.

We first tested the effect of exogenous NaCl treatment on soybean seed germination. The germination analysis showed that NaCl delays the seed germination processes in cultivar ND-12 (**Figures 1A,B**). The germination rate of the NaCl-treated soybean seeds was about two to three-fold less than the control (CK) during the germination processes (**Figure 1B**). Consistently, the analysis of several post-germinative growth parameters, including the length of radicle (**Figure 1E**) and the fresh weights of germinated seeds (**Figure 1F**), also supported that the delayed-germination phenotype was caused by the NaCl treatment (**Figures 1A,B**). To further confirm the inhibition effect of NaCl on soybean seed germination processes, we tested another cultivar, HD-19, which is genetically distinct from the ND-12 cultivar. The results revealed that NaCl treatment also inhibited HD-19 seed germination processes (**Figures 1C,D**), and the post-germinative growth analysis showed that post-germinative growth was also reduced by NaCl (**Figures 1G,H**). Exogenous NaCl application had a remarkable inhibitory effect on radicle length and the fresh weights of germinated seeds (**Figures 1G,H**).

Subsequently, we also analyzed the germination rate of the black soybean cultivar, C-103. The results revealed that NaCl application inhibited C-103 seed germination processes (**Supplementary Figures S1A,B**), and significantly reduced the radicle length and fresh weights of germinated seeds (**Supplementary Figures S1C,D**), which was similar to the analysis of HD-19 and ND-12 cultivars. These results demonstrated that exogenous NaCl treatment delays soybean seed germination processes.

### NaCl Application Caused Oxidative Stress during Soybean Seed Germination

**FIGURE 2:**
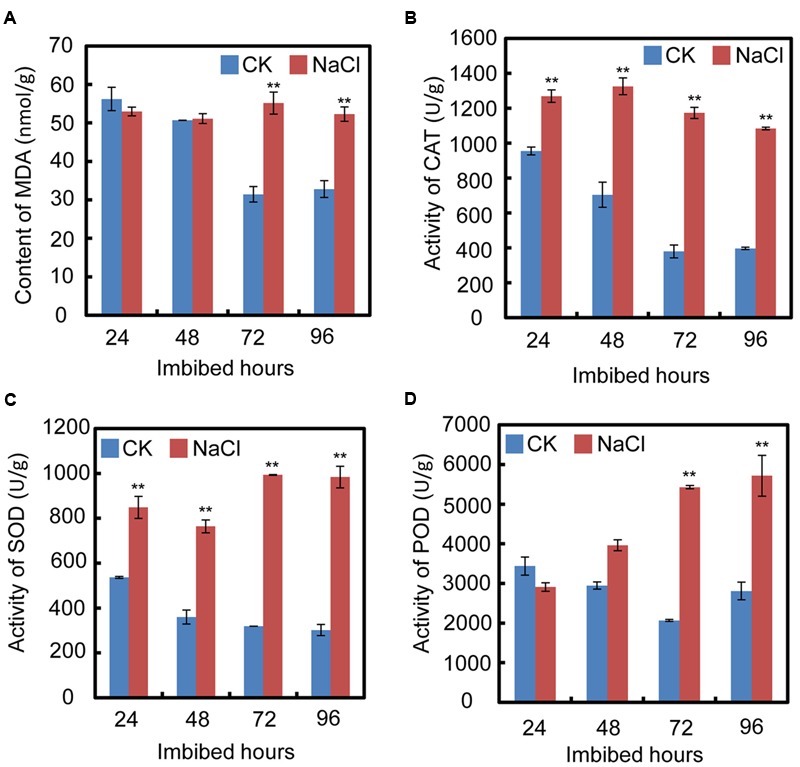
Quantification of MDA (malondialdehyde) content, activities of superoxide dismutase (SOD), catalase (CAT), and peroxidase (POD) during soybean seed germination after NaCl application. **(A)** Effect of NaCl on change of MDA content during seed imbibition. **(B–D)** Activities of CAT, SOD, and POD after exogenous NaCl treatment during soybean seed germination. Soybean cultivar ND12 seeds were employed and the concentration of NaCl used was 150 mM. Student’s *t*-test assay was employed for statistical analysis, and ^∗∗^ difference is significant at the 0.01 level.

In order to test the cell oxidative status after treatment with exogenous NaCl, oxidative stress parameters, including malondialdehyde (MDA) content, and catalase (CAT), superoxide dismutase (SOD), and peroxidase (POD) activities, were investigated during the seed germination processes. The results show that after exogenous NaCl treatment, the MDA content significantly increased, especially from 72 h to 94 h after imbibition (**Figure 2A**), which suggested that, during soybean seed germination and post-germinative growth, NaCl treatment increased the toxic stress in cells and inhibited both processes. Furthermore, the CAT, SOD, and POD activities also increased after NaCl application (**Figures 2B–D**). The increase in those enzymes suggested that NaCl treatment caused oxidative stress in cells during seed germination.

### NaCl Enhances ABA Biogenesis, but Impairs GA Biosynthesis

Previous studies have demonstrated that ABA and GA play key roles in seed germination (Graeber et al., 2012; Shu et al., 2016b). Therefore, we further investigated the missing links between the inhibitory effect of NaCl on soybean seed germination and the effects on the GA and ABA pathways. Consequently, we explored the transcription patterns of ABA and GA biogenesis and signaling genes in soybean seeds during the imbibition process using a time-course analysis. Then we further analyzed the ABA and active GA content during seed imbibition after NaCl treatment.

**FIGURE 3:**
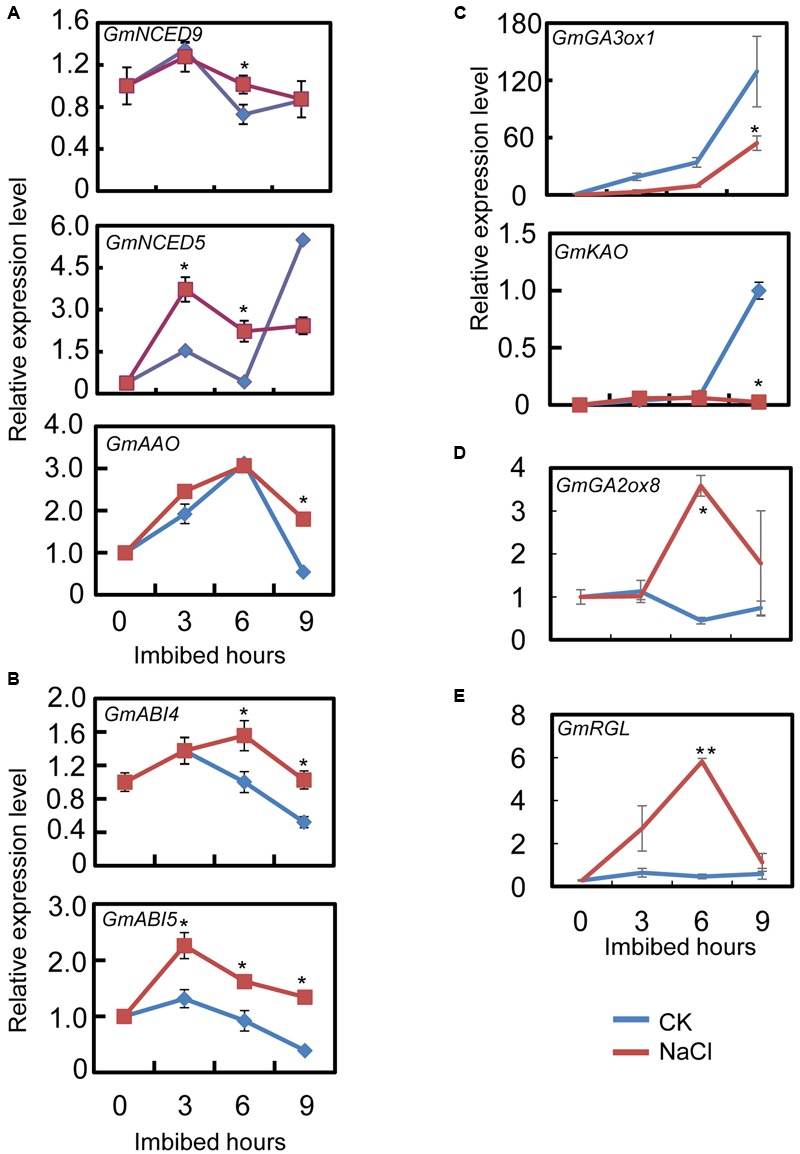
Gene transcription pattern analysis during the course of seed imbibition. Soybean cultivar ND12 dry seeds and imbibed seeds (0, 3, 6, 9 h after imbibition) were used for total RNA extraction. Three biological replications were performed. **(A)** Abscisic acid (ABA) biosynthesis genes, *GmNCED9, GmNCED5*, and *GmAAO*. **(B)** ABA signaling positive regulator genes, *GmABI5 and GmABI4.* **(C)** Gibberellin (GA) biosynthesis genes, *GmGA3ox1 and GmKAO.* **(D)** GA inactivate gene, *GmGA2ox8.* **(E)** negative regulator gene in GA signaling transduction pathway, *GmRGL*. Three replications were performed and similar results were obtained. The concentration of NaCl used was 150 mM. Student’s *t*-test assay was employed for statistical analysis, and asterisks indicate statistically significant differences from CK at different time points (^∗^*P* < 0.05; ^∗∗^*P* < 0.01). Primer pairs of the marker genes are listed in Supplementary Table S1.

The qRT-PCR results showed that the transcript level of the ABA biosynthesis genes: *GmNCED9, GmNCED5*, and *GmAAO*, were significantly up-regulated by NaCl-treatment during seed imbibition compared to the CK (**Figure 3A**). The NaCl treatment enhanced *GmNCED9* expression at 6 h after imbibition, whereas NaCl-induced *GmNCED5* transcription was also detected at 3 and 6 h after sowing (**Figure 3A**). Higher *GmAAO* transcription levels were maintained, especially at 9 h after imbibition (**Figure 3A**). We next investigated the transcription levels of genes involved in the ABA signaling pathway. **Figure 3B** shows that the exogenous NaCl application considerably enhanced ABA signaling by increasing *GmABI4* and *GmABI5* transcription because *GmABI4* and *GmABI5* are key positive regulators in the ABA signaling pathway (Shu et al., 2016b). Taken together, the transcription pattern analysis revealed that ABA content and signaling were possibly enhanced by exogenous NaCl treatment during soybean seed germination.

Subsequently, we further examined the expression pattern of the key genes involved in GA biogenesis and the signaling pathways. The qRT-PCR assay showed that after NaCl treatment the level of GA biosynthesis genes *GmGA3ox1* and *GmKAO* decreased, especially at 9 h after sowing, compared to the CK (**Figure 3C**). On the other hand, GA inactive gene *GmGA2ox8* expression increased during seed imbibition (**Figure 3D**). Furthermore, *GmRGL* transcription, a gene that belongs to the DELLA family and represses seed coat rupture during seed germination (Piskurewicz and Lopez-Molina, 2009), was up-regulated considerably during imbibition after NaCl-treatment (**Figure 3E**). Taken together, these analyses revealed that exogenous NaCl probably negatively regulated GA biogenesis and signaling during soybean seed germination.

**FIGURE 4:**
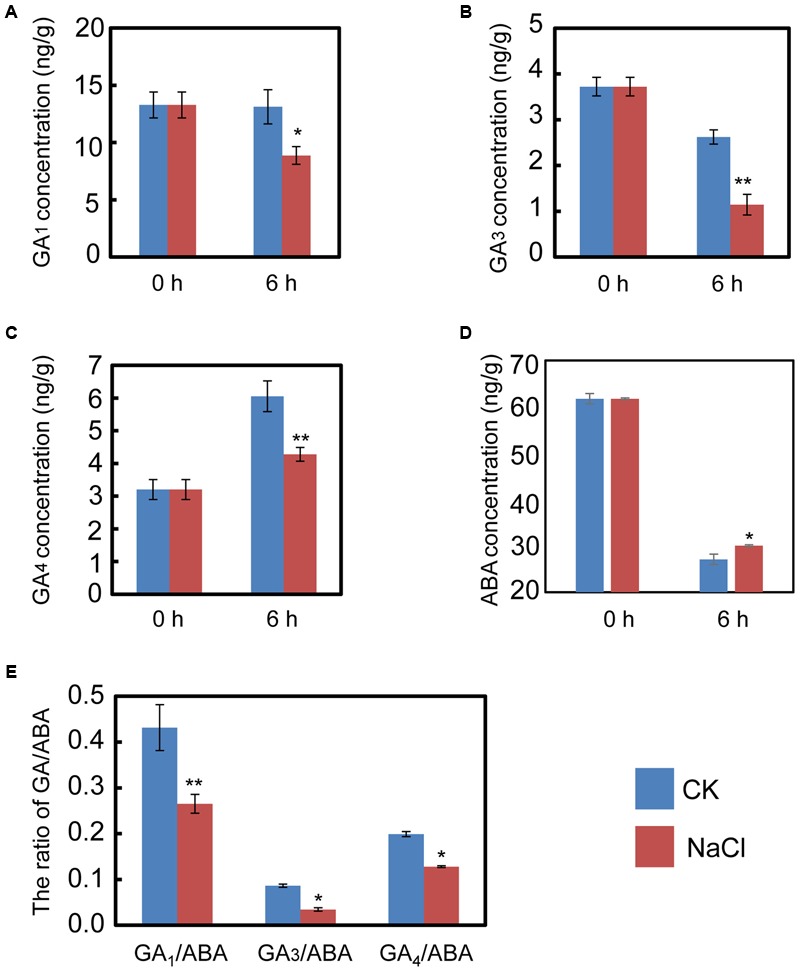
NaCl decreases the ratios between active GA and ABA, including GA_1_/ABA, GA_3_/ABA, and GA_4_/ABA. Soybean seeds were incubated at 25°C and treated with NaCl. Equivalent ultrapure water was added as control (CK). Dry seeds and 6-h imbibed seeds were used to determine the concentration of endogenous active GA_1_, GA_3_, and GA_4_. The results showed that NaCl treatment decreases active GA_1_ **(A)**, GA_3_ **(B)**, and GA_4_ **(C)**, while ABA content was increased after NaCl application **(D)**. The ratios of GA_1_/ABA, GA_3_/ABA, and GA_4_/ABA **(E)** were also shown. The concentration of NaCl used was 150 mM. Student’s *t*-test assay was employed for statistical analysis, and ^∗^ difference is significant at the 0.05 level, ^∗∗^ difference is significant at the 0.01 level.

The qRT-PCR data described above hinted us that NaCl may mediate ABA and GA biogenesis by regulating the expression of the metabolic genes for both phytohormones. Consequently, the endogenous ABA and active GA levels during imbibition were quantified. The results indicated that NaCl significantly decreased active GA_1_, GA_3_, and GA_4_ levels (**Figures 4A–C**). In contrast, the ABA content in NaCl-treated soybean seed during imbibition remarkably increased (**Figure 4D**), which meant that the GA_1_/ABA, GA_3_/ABA, and GA_4_/ABA ratios were down-regulated (**Figure 4E**). The active GA/ABA ratio is a key factor during seed germination (Finkelstein et al., 2008; Shu et al., 2016a,b). Therefore, the measurement of both ABA and GA supported the transcription analysis and the phenotype description. Altogether, all the results suggest that exogenous NaCl treatment positively regulates ABA biogenesis, but negatively mediates GA biosynthesis.

### FLUN Partially Rescues the Delayed-Germination Phenotype of NaCl-Treated Seeds

To further confirm that NaCl enhances ABA biogenesis while impairs GA biogenesis during soybean seed germination, we next tested the responsiveness of soybean seeds to FLUN, an ABA biosynthesis inhibitor, and paclobutrazol (PAC), a GA biosynthesis inhibitor, during seed imbibition.

**FIGURE 5:**
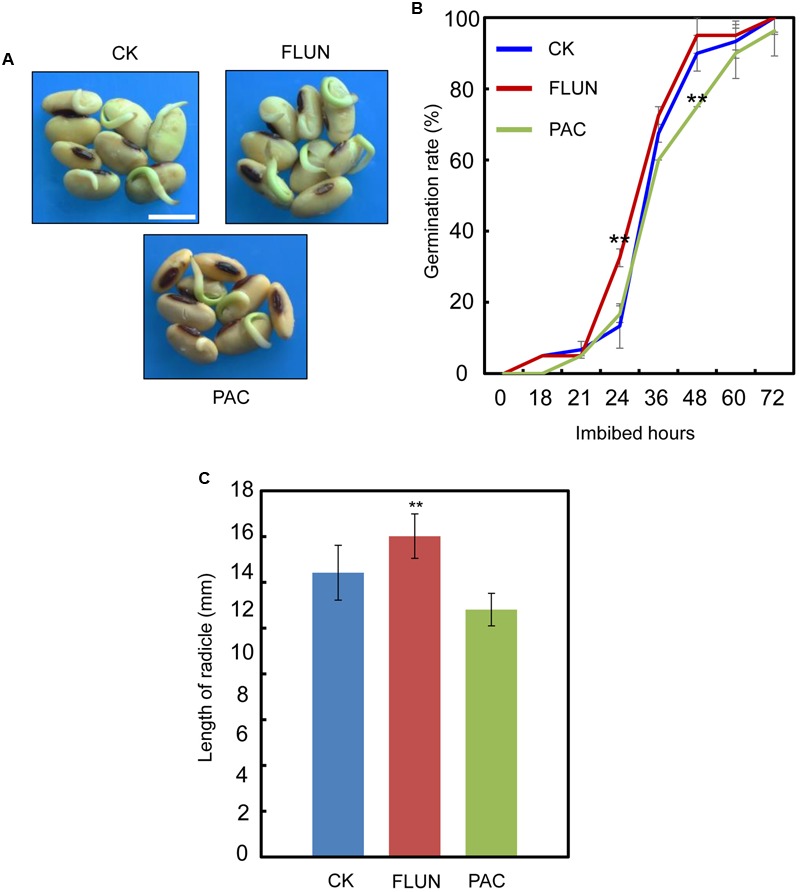
Gibberellin (GA) biogenesis inhibitor PAC, and ABA biogenesis inhibitor fluridone (FLUN) regulates soybean seed germination oppositely. **(A,B)** Soybean seeds (cultivar ND12) were incubated on two layers of filter paper in Petri dishes at 25°C and treated with 100 nM FLUN and10 μM PAC. Quantitative analysis of germination rates is shown in **(B)**. Radicle length **(C)** of germinated seeds were measured under FLUN and PAC treatments and CK. The average length and fresh weight of four repeats are shown. Student’s *t*-test assay was employed for statistical analysis. ^∗∗^ Difference is significant at the 0.01 level, while ^∗^ difference is significant at the 0.05 level. Bar = 10 mm.

**FIGURE 6:**
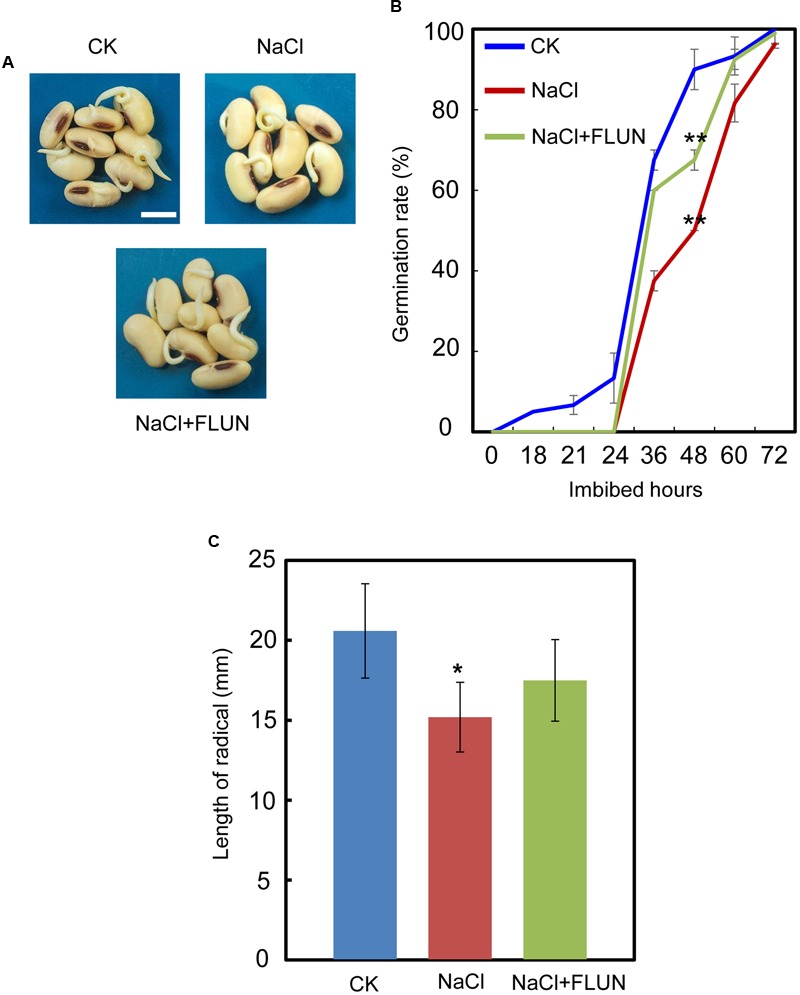
Fluridone partially rescues the repressed-germination phenotype resulting from NaCl stress. **(A)** and **(B)** Soybean cultivar ND12 seeds were incubated on two layers of filter paper in Petri dishes at 25°C and treated with 150 mM NaCl and 100 nM FLUN + 150 mM NaCl, respectively. Quantitative analysis of germination rates is shown in **(B)**. The representative images (36 h after sowing) are shown **(A)**. Radicle length of germinated seeds were measured **(C)**. Student’s *t*-test assay was employed for statistical analysis. ^∗∗^ Difference is significant at the 0.01 level, while ^∗^ difference is significant at the 0.05 level. When performed the statistical analysis, the data of NaCl treatment were used as the baseline in **(B)**, while the data of CK were used as baseline in **(C)**. Bar = 10 mm.

**FIGURE 7:**
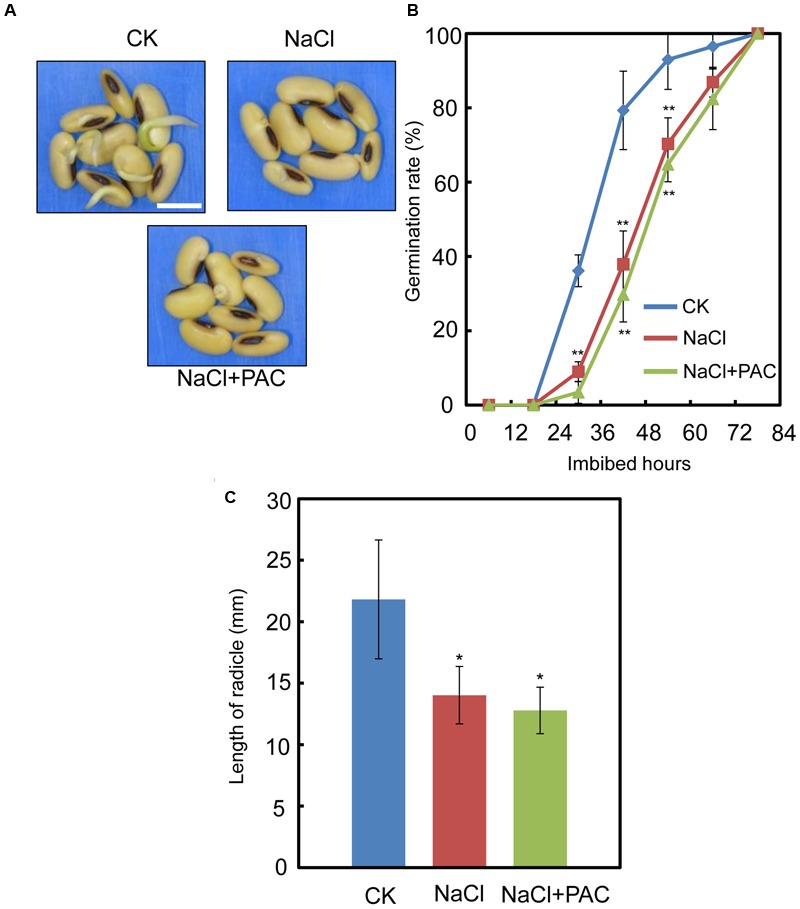
The effect of PAC+NaCl combination treatment on soybean seed germination, Soybean cultivar ND12 seeds were used. **(A–B)** Soybean seeds were incubated on two layers of filter paper in Petri dishes at 25°C and treated with 150 mM NaCl and 100 nM PAC + 150 mM NaCl, respectively. Quantitative analysis of germination rates is shown in **(B)**. The representative images (36 h after sowing) are shown **(A)**. Radicle length of germinated seeds were measured **(C)**. The CK data were used as baseline when performed the statistical analysis. Student’s *t*-test assay was employed for statistical analysis. ^∗∗^ Difference is significant at the 0.01 level, while ^∗^ difference is significant at the 0.05 level. Bar = 10 mm.

We also investigated the effect of the FLUN and PAC treatments on soybean seed germination. The results showed that FLUN promotes soybean seed germination, whereas PAC delays imbibition and germination (**Figures 5A,B**). The effects on post-germination growth parameters, such as radicle length (**Figure 5C**), were also similar to the germination results. The NaCl-treatment clearly promotes ABA biosynthesis during soybean seed germination, and FLUN blocks the ABA biosynthesis pathway. Therefore, we hypothesized that FLUN may be able to rescue the delayed-germination phenotype caused by enhanced ABA biogenesis. The germination analysis showed that FLUN could partially restore the delayed-germination phenotype seen when soybean seeds are treated with NaCl (**Figures 6A,B**). As shown in **Figure 6B**, although the seed germination rate of NaCl treatment was similar to NaCl+FLUN treatment within 24 h imbibition, the germination rate of NaCl+FLUN was higher than that of NaCl, but lower than that of CK between 36 to 60 h of imbibition (**Figures 6B**). This indicates that exogenous application of FLUN partially rescued salt stress inhibition of seed germination, which was also observed in **Figure 6C** based on radicle length. In other words, some other factors might be involved in salt stress-induced delay of seed germination (**Figures 6C**). On the other hand, we also found that NaCl+PAC mimics the inhibition effect caused by NaCl treatment (**Figures 7A,B**), and the post-germinative growth parameters further supported this (**Figure 7C**). Taken together, these results demonstrated that NaCl delays the soybean seed germination process by promoting ABA biosynthesis while impairing GA biosynthesis.

## Discussion

The large tracts of saline land that exist worldwide significantly constrain the development of sustainable agriculture, and on the other hand, they could also be used to improve food security after reclamation. However, the modification of salt-affected soils is very expensive and time-consuming. Consequently, increased plant salt resistance during different growth stages may provide a lower cost alternative for modern agriculture. Therefore, potential plant growth regulators should be explored further. In this study, we dissected the mechanisms through which salt repress soybean seed germination, and further showed that FLUN might be a potential plant growth regulator that could be used to promote soybean seed germination under salinity stress.

It is well-known that salt inhibits soybean seed germination and post-germinative growth, which eventually leads to decreases in yield (Hamayun et al., 2015). Previous investigations and the present study showed that NaCl treatment caused cellular oxidative stress (**Figure 2**; Liang et al., 2003; Zhu, 2016; Farhangi-Abriz and Torabian, 2017), and the level of cellular oxidative pressure is further associated with the activity of seed germination (Ma et al., 2017). In the past decade, soybean salt tolerance-related quantitative trait loci and some key proteins have been identified over the past few decades (Xu et al., 2011; Guan et al., 2014; Kan et al., 2015). However, the detailed molecular mechanisms underlying the inhibition-effect of salt on soybean seed germination and post-germinative growth are not fully understood, especially the relationship between salt effects and the ABA and GA pathways. In this study, the seed germination phenotype description (**Figure 1** and **Supplementary Figure S1**), the biochemical analysis (**Figure 2**), the gene transcription pattern analysis (**Figure 3**), and the phytohormones measurement results (**Figure 4**) all demonstrated that NaCl stress positively regulated ABA biogenesis, but negatively mediated the GA biosynthesis pathways. This led to a decrease in the GA and ABA ratios (including GA_1_/ABA, GA_3_/ABA, and GA_4_/ABA). Importantly, we further have shown that FLUN might be a potential plant growth regulator that could promote soybean seed germination under salinity stress (**Figure 6**).

FLUN blocks ABA biosynthesis and disrupts plant photosynthesis (Stetsenko et al., 2015). It is also an aquatic herbicide that is usually used to control invasive plants (Gamble and Mullet, 1986; Sun et al., 2012). However, recent studies have revealed that FLUN is also involved in other processes, such as the regulation of plant drought stresses responses, plant development control, and even human disease control. It is well known that both heavy metals (including Cu^2+^, Cd^2+^, and Hg^2+^) and heat stress stop maize seed germination, but FLUN can significantly alleviate thermodormancy and rescue the delayed-germination phenotype caused by heavy metals (Deng et al., 2016). Furthermore, applying FLUN to tobacco leaves can promote *NtEXGT* expression and further increases plant drought resistance (Kuluev et al., 2017). It has also been recently demonstrated that FLUN stimulated *Vellozia* sp. seed germination in the dark (Vieira et al., 2017). Furthermore, FLUN has potential as a candidate drug for the treatment of acute neosporosis *in vivo* (Ybanez et al., 2016) and as a new anti-inflammatory drug (Magnone et al., 2013). These recent studies have demonstrated that FLUN possesses many different biological functions in both plants and animals.

Salinity inhibits soybean development across the whole life-cycle, which finally leads to decreased yields. Seed germination and emergence in the field are key crop yield determinants. However, large parts of the world are covered in saline soil, which significantly limits soybean seed germination and yield. Therefore, enhancing soybean seed germination under salt stress conditions will be of major benefit to agriculture worldwide. In this study, we showed that applying FLUN can rescue the delayed-germination phenotype caused by NaCl treatment. Consequently, FLUN can be a potential plant growth regulator that could be used to promote soybean seed germination under salt stress. Last but not least, it is noted that NaCl treatment caused oxidative stress in soybean seed cells (**Figure 2**), thus it is valuable to test whether and how FLUN enhances soybean seed germination under salt stress through activation of the cellular antioxidant systems.

## Materials and Methods

### Plant Materials and Growth Condition

Soybean cultivars employed in the present investigation were Hedou-19 (HD-19), Nandou-12 (ND-12) and C-103. HD-19 and ND-12 (yellow seed coat in color) and C-103 (black seed coat in color) are the prevailing soybean cultivars in Southwestern China, and are breeded by Nanchong Academy of Agricultural Sciences, Sichuan Province, China. These three genotypes are distinct regards to the genetic background. These variates were grown in Science and Technology Campus, Sichuan Agricultural University, and were harvested at the same time. The elite soybean seeds were chosen for further investigation.

### Seed Germination Phenotypic Analysis

For each petri dishes, 20 soybean seeds were incubated on two layers of medium-speed qualitative filter papers in Petri Dishes (diameter is 9 cm). Then, 11 mL distilled water, NaCl (at 150 mM), 100 nM FLUN or 10 μM paclobutrazol (PAC) were add to each plate according to the experiment requirements. Four replications (Petri Dishe) were performed. The plates were incubated at 25°C in incubator (Sanyo Versatile Environmental Teat Chamber MLR-350H). Germination rates were considered when the radicle broke through the seed coat. The germination percentages were counted at with time-course analysis.

After 60 h of imbibition, the radicles will be cut off, and the length and fresh weight of radicles per 20 seeds were measured. The Image J software was employed to measure the length of radicles. For each germination test, three experimental replications were performed. The average germination percentage ± SE (standard error) of triplicate experiments was calculated. NaCl (product number 7647-14-5), FLUN (product number 45511), and PAC (product number 33371) were ordered from Sigma–Aldrich Company Ltd., United States.

### Quantification of Activities of SOD, CAT, and POD and MDA Content

After NaCl treatment, MDA content and the activities of SOD, CAT, and POD were detected during soybean seed germination processes with time-course pattern. The method published elsewhere were employed for the quantification (Yang et al., 2010).

### Gene Expression Analysis

Imbibition seeds were collected at different time points after sowing and frozen in liquid nitrogen quickly. Total RNA preparation, first-strand cDNA synthesis and qRT-PCR assay were performed as our previously described (Shu et al., 2013). Quantitative PCR was performed using the SsoFast^TM^ EvaGreen Supermix (Bio-Rad). Each 10 μL reaction comprised 2 μL template, 5 μL SsoFast^TM^ EvaGreen Supermix, 0.5 μL (10 μM) of each primer and 2 μL Dnase-free ddH_2_O. The transcription pattern of genes involved in ABA/GA biosynthesis and signaling transduction pathways including *GmNCED9, GmNCED5, GmAAO, GmABI4, GmABI5*, and *GmGA3ox1, GmKAO, GmGA2ox8*, and *GmRGL* were investigated. Three biological replicates were performed for each experiment. Primer sequences for qRT-PCR are shown in Supplemental Table S1.

### Quantification of ABA in Soybean Seeds

For analysis of ABA content in soybean seeds, the previously protocol we used (Shu et al., 2013) were employed in this study. Firstly, the seeds were ground to powder in liquid nitrogen, and 300 mg of seeds powder was homogenized and extracted for 24 h in methanol containing D6-ABA (OIChemIm Co. Ltd.) as an internal standard. Purification was performed with an Oasis Max solid phase extract cartridge (Waters) and eluted with 5% formic acid in methanol. Subsequently, the elution was dried and reconstituted, and it was then injected into a liquid chromatography–tandem mass spectrometry system consisting of an Acquity ultra performance liquid chromatograph (Acquity UPLC; Waters) and a triple quadruple tandem mass spectrometer (Quattro Premier XE; Waters). Three biological replications were performed.

### Quantification of Endogenous Gibberellins

The endogenous gibberellins were determined according to our method described previously (Chen et al., 2011). Soybean seeds (400 mg) were frozen in liquid nitrogen, ground to fine powder, and extracted with 80% (v/v) methanol. GA d_2_ isotope standards were added to plant samples before grinding. The crude extracts were purified by reversed-phase solid-phase extraction, ethyl ether extraction, and derivatization. The resulting mixture was injected into capillary electrophoresis-mass spectrometry (CE-MS) for quantitative analysis. Three biological replications were performed.

### Statistical Analysis

The data including germination rates, fresh weight and radicle length of germinated seeds, gene expression data, and phytohormones quantification results were analyzed using Student’s *t*-test. Different baselines were employed according to different requirements, and the detailed information was presented in the figure legends.

## Author Contributions

KS designed the research. YQ, FC, YM, XL, HS, WZ, JDi, JDu, JL, FY, QW, WL, TY, XW, and YF performed the experiments. KS and WY analyzed the data and wrote the manuscript.

## Conflict of Interest Statement

The authors declare that the research was conducted in the absence of any commercial or financial relationships that could be construed as a potential conflict of interest.

## Supplementary Material

FIGURE S1 NaCl inhibits soybean seed germination. Cultivars C-103 seeds was incubated on two layers of filter paper in Petri dishes. The concentration of NaCl used was 150 mM, and the equivalent ultrapure water was employed as control (CK). **(A)** The representative images (36 h after sowing) are shown. **(B)** Quantitative analysis of germination rates is shown. **(C,D)** Radicle length and fresh weight of germinated soybean seeds were measured. Bar = 10 mm. The average percentages of four repeats ±standard error were shown. Student’s *t*-test assay was employed for statistical analysis. ^∗∗^ Difference is significant at the 0.01 level.
