# Supplementary figures and images for: Salt Stress Represses Soybean Seed Germination by Negatively Regulating GA Biosynthesis While Positively Mediating ABA Biosynthesis
Source: Front Plant Sci. 2017 Aug 10;8:1372. doi: 10.3389/fpls.2017.01372 (PMC5554363; doi:10.3389/fpls.2017.01372)

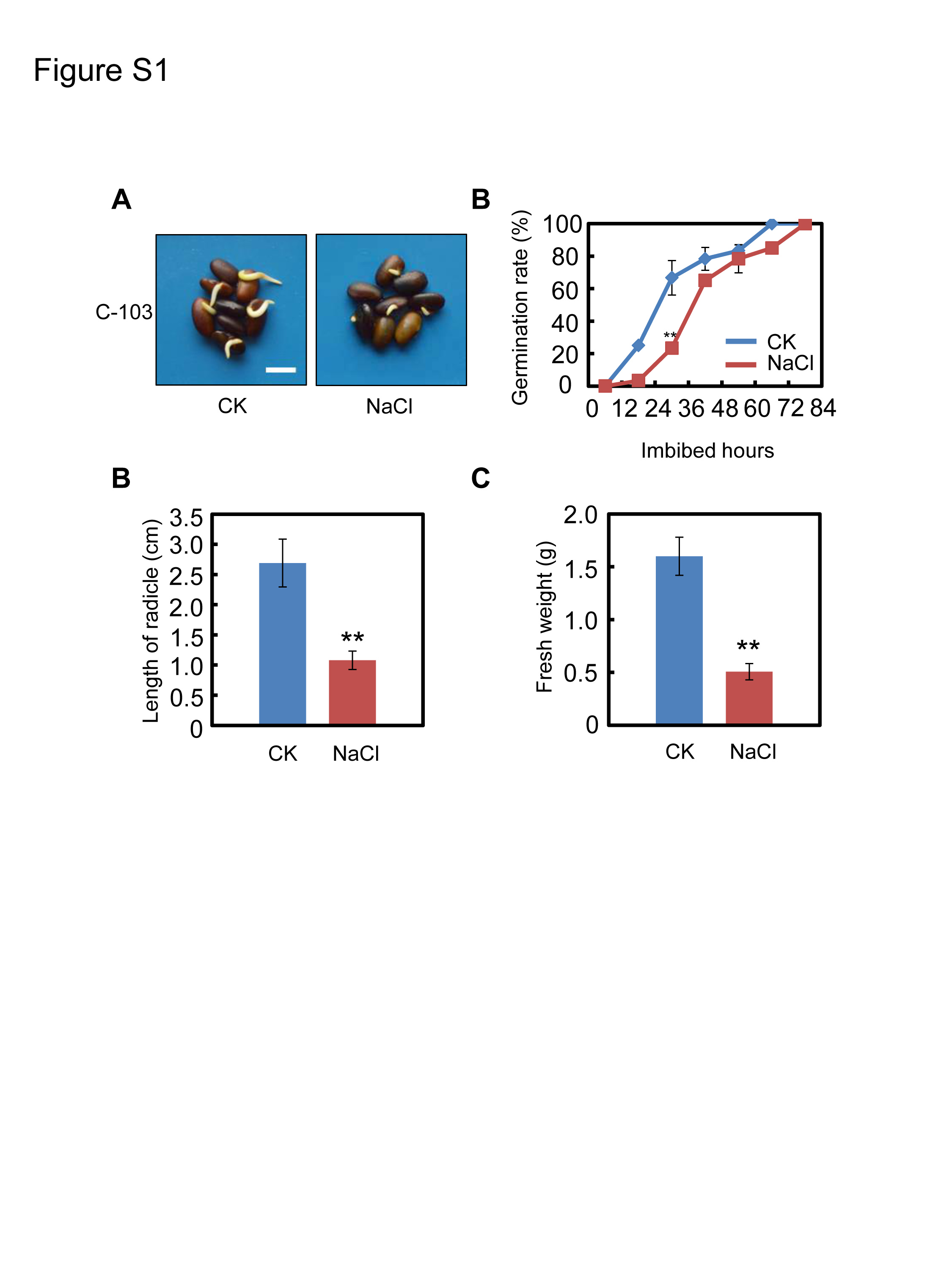

Supplement: FIGURE S1 — NaCl inhibits soybean seed germination. Cultivars C-103 seeds was incubated on two layers of filter paper in Petri dishes. The concentration of NaCl used was 150 mM, and the equivalent ultrapure water was employed as control (CK). (A) The representative images (36 h after sowing) are shown. (B) Quantitative analysis of germination rates is shown. (C,D) Radicle length and fresh weight of germinated soybean seeds were measured. Bar = 10 mm. The average percentages of four repeats ±standard error were shown. Student’s t-test assay was employed for statistical analysis. ∗∗ Difference is significant at the 0.01 level. [file Image_1.JPEG]
