# Supplementary material for: Salt Stress Represses Soybean Seed Germination by Negatively Regulating GA Biosynthesis While Positively Mediating ABA Biosynthesis
Source: Front Plant Sci. 2017 Aug 10;8:1372. doi: 10.3389/fpls.2017.01372 (PMC5554363; doi:10.3389/fpls.2017.01372)
Supplement: Supplementary file 2 [file Table_1.DOCX]

**Supplemental Table 1.** Primers for qRT-PCR in this study.

| Primers | Sequence |
| --- | --- |
| *qRT-GmGA3ox1Fw* | CTCGCATCTCTTCCTTCTTCC |
| *qRT-GmGA3ox1Rev* | AATCCAACATCAGCCACATCAG |
| *qRT-GmABI2Fw* | CATATCAACAATCTAATCTC |
| *qRT-GmABI2Rev* | ACCTAACACTTCTACAC |
| *qRT-GmABI4Fw* | AATCAACACAACACAACA |
| *qRT-GmABI4Rev* | ACCAAAACATCCATAC |
| *qRT-GmABI5Fw* | CATTCCACACATCT |
| *qRT-GmABI5Rev* | TTTCTCTTCACTTCCA |
| *qRT-GmKAOFw* | TTAAAAATAATTT |
| *qRT-GmKAORev* | TCTCTTATTTTTAATAA |
| *qRT-GmNCED6Fw* | CTACACACAAAAA |
| *qRT-GmNCED6Rev* | CAAATAAAAAAC |
| *qRT-GmNCED5Fw* | CTACACACAAAAA |
| *qRT-GmNCED5Rev* | CAAATAAAAAAC |
| *qRT-GmNCED9Fw* | CACACTCAATTCAACACTT |
| *qRT-GmNCED9Rev* | TAACACAATCCACCACCTT |
| *qRT-GmActinFw* | CTATCTCACTATCCCTCAT |
| *qRT-GmActinRev* | ACCTTCCAATCCACATCTTT |
